# Knockdown of LINC01694 inhibits growth of gallbladder cancer cells via miR-340-5p/Sox4

**DOI:** 10.1042/BSR20194444

**Published:** 2020-04-24

**Authors:** Lei Liu, Yuexiang Yan, Guanyu Zhang, Chengxue Chen, Weihong Shen, Peixiang Xing

**Affiliations:** 1 Department of General Surgery, Hanting District People’s Hospital of Weifang, Weifang, Shandong Province, China; 2 General Branch 2, Weifang Yidu Central Hospital, Weifang, Shandong Province, China; 3 School of Medicine, Nanchang Univeristy, Nanchang, Jiangxi Province, China; 4 Dingtao District People’s Hospital of Heze, Heze, Shandong Province, China; 5 Department of Clinical Laboratory, Qilu Hospital, Shandong University, Jinan 250012, Shandong Province, China

**Keywords:** ceRNA, gallbladder cancer, LINC01694, miR-340-5p, prognosis, Sox4

## Abstract

**Purpose:** The indispensable role of long non-coding RNAs (lncRNAs) in tumorigenesis has been increasingly reported. In the present study, LINC01694 was found to regulate the proliferation, invasion, as well as apoptosis in gallbladder cancer (GBC) cells through sponging miR-340-5p.

**Methods:** LINC01694 level in GBC cells was quantified by quantitative real-time polymerase chain reaction (qRT-PCR). The proliferation, invasion, and apoptosis were determined by Cell Counting Kit-8 (CCK-8), Transwell, and flow cytometry, respectively. The expression of Sry-related high-mobility group box 4 (Sox4) was detected by Western blot (WB). The interaction between LINC01694 and miR-340-5p was measured by dual-luciferase reporter (DLR) assay, RNA immunoprecipitation (RIP) test, and RNA pull-down. Tumor formation was examined by *in vivo* experiment.

**Results:** qRT-PCR illustrated that cancerous tissues had higher LINC01694 than normal tissues. Survival analysis demonstrated that the prognosis of patients with high LINC01694 was significantly poorer than those with low LINC01694. Down-regulation of LINC01694 slowed down the proliferation and invasion in GBC cells and accelerated the apoptosis. DLR assay indicated that LINC01694 elevated Sox4 expression by regulating miR-340-5p. LINC01694 functioned as miR-340-5p sponge to inhibit Sox4 expression.

**Conclusion:** LINC01694 level is elevated in GBC by regulating miR-340-5p/Sox4 axis, which indicates the poor prognosis of the patients.

## Introduction

Gallbladder cancer (GBC) is a prevalent biliary duct tumor and the sixth most common gastrointestinal cancer in the world [1,2]. In a tumor epidemiological survey, more than 150000 each new and dead cases of GBC patients were reported in 2018 [3]. Although the morbidity and mortality are relatively low compared with other digestive tract tumors, the 5-year survival remains extremely poor. Generally, GBC is diagnosed at an advanced stage due to the occult early symptoms and the lack of diagnostic markers [4,5]. Although improvements can be made by radiotherapy and chemotherapy, the survival and prognosis of advanced patients is still disappointing. Therefore, seeking potential diagnostic and molecular targets for GBC is of great significance [6].

Non-coding RNA (ncRNA) has been a hot topic in various disciplines, especially in oncology, which plays a vital role in tumor progression [7,8]. Long ncRNA (lncRNA), the most well-known ncRNA [9], was once considered a metabolic ‘waste’ in transcription process for its incapability to encode proteins directly [10]. However, recently, it has been found to exhibit an important regulatory effect in various stages of cancers [11–13]. Moreover, it regulates tumor growth, cell proliferation, invasion, and metastasis, and is associated with tumor recurrence [14]. LINC01694 is an member of lncRNAs located on human chromosome 21q22.3, and its role in tumors has never been reported previously. In the present study, we screened the potential lncRNAs in GBC through Gene Expression Omnibus (GEO), and noticed the high expression of LINC01694, which suggested that LINC01694 may be a potential marker of GBC. Moreover, we found a close association between LINC01694 and Sry-related high-mobility group box 4 (Sox4) using prediction tools. Sox4 is a regulatory protein regulating embryonic development, and cell death and apoptosis [15]. It has also been reported to participate in the growth and metastasis of GBC.

Therefore, the value and relevant mechanism of LINC01694 in GBC were analyzed by basic experiments to provide potential diagnostic and molecular targets.

## Methods

### GEO dataset analysis

Logging in https://www.ncbi.nlm.nih.gov/gds, GSE74048 and GSE104165 screened were analyzed using GEO2R included in the GEO. A total of six samples from GSE74048, including three control samples (GSM1908732, GSM1908731, GSM1908733) and three tumor samples (GSM1908730, GSM1908728, GSM1908729), were collected to identify differential lncRNAs in GBC. Meanwhile, a total of 48 samples from GSE104165, including 8 control samples (GSM2791013, GSM2791012, GSM2791011, GSM2791008, GSM2791007, GSM2791010, GSM2791014, GSM2791009) and 40 tumor samples (GSM2791006, GSM2790983, GSM2791001, GSM2790984, GSM2790988, GSM2790973, GSM2791004, GSM2790997, GSM2790970, GSM2790999, GSM2790967, GSM2790987, GSM2790981, GSM2790975, GSM2790979, GSM2791002, GSM2790980, GSM2791003, GSM2790976, GSM2791000, GSM2790998, GSM2790985, GSM2790977, GSM2790971, GSM2790993, GSM2790968, GSM2790991, GSM2790996, GSM2790992, GSM2790995, GSM2790969, GSM2790989, GSM2790972, GSM2790990, GSM2790982, GSM2790986, GSM2790994, GSM2790974, GSM2791005, GSM2790978), were collected to identify differential miRs.

### Sample collection

Forty patients with GBC who were diagnosed and treated in Qilu Hospital of Shandong University from February 2013 to June 2015 were enrolled as the patient group. Cancerous and adjacent tissues obtained during the surgery were examined at the pathology department. The samples were stored at −80°C. Peripheral blood (5 ml) from patients and normal individuals were collected, left standing for 30 min, and centrifuged at 3000 rpm for 10 min to obtain the sera. Another 30 healthy individuals were enrolled as the control group. The patients recruited received no anti-cancer treatment before the present study. All patients met the criteria of tumor-node-metastasis (TNM) cancer staging system issued by the American Joint Committee on Cancer (AJCC) (7th edition) [16]. All patients signed the informed consent and cooperated with the follow-up. Patients with an expected survival time of less than 1 month were excluded. The present study was approved by Medical Ethics Committee in Qilu Hospital of Shandong University. The follow-up ended in June 2019 and the survival was recorded by telephone and outpatient electronic medical records.

### Cell culture and transfection

GBC cell lines (GBC-SD, SGC-996, NOZ, OCUG, and EHGB-1) and control cell line (293T) were recruited (Shanghai Institute of Biochemistry and Cell Biology, Chinese Academy of Sciences). The cells were cultured in Dulbecco’s modified Eagle’s medium (DMEM, Gibco BRL, U.S.A.) with 10% fetal bovine serum (FBS) and penicillin/streptomycin (Invitrogen, U.S.A.) under a humid environment (5% CO_2_ and 37°C). Transfection was performed with Lipofectamine 2000 (Invitrogen, U.S.A.). After covering the whole monolayer, the cells were planted in a 24-well plate (approximately 1 × 10^5^ cells/well). Transfection was carried out on the following day when the cells reached approximately 80% confluence. miR transfection: 400 μg/well plasmid, 1 μl RNA, 2 μl lipo2000; lncRNA transfection: 800 ng/well plasmid, 1 μl RNA, 2 μl lipo2000. LINC01694 inhibitor (si-LINC01694) and Sox4 inhibitor (si-Sox4) were constructed, and LINC01694 overexpression vector (pcDNA-3.1-LINC01694) and blank vector (pcDNA-3.1-NC) were established with PcDNA-3.1. In addition, the sequences of miR-340-5p mimics (miR-340-5p-mimics), inhibitor (miR-340-5p-inhibit), and blank sequence (miR-NC) were synthesized by Shanghai Sangon Biology Co., Ltd. The construction of recombinant lentivirus vector for nude mouse tumorigenicity were as follows: 100 μl virus and 3 μl polybrene were added in 800 μl DMEM containing 10% FBS, and then the medium was moved to a six-well plate. After 4 h of culture, 3 μl polybrene and 1 ml DMEM containing 10% FBS were added to the plate. Twenty-four hours later, the medium was replaced with 2–3 ml complete medium. Cells were harvested after 48 h and infection efficiency was determined by quantitative real-time polymerase chain reaction (qRT-PCR). The cell lines with stably overexpressed (HBLV-h-LINC01694) and knockdown (sh-LINC01694) LINC01694 were used in the follow-up experiment as well as the control cell line.

### Cell proliferation

The Cell Counting Kit-8 (CCK-8) (Beyotime Biotechnology, Shanghai, China was employed to determine cell growth. Cells transfected for 48 h were prepared into suspension and placed in a 96-well plate, then 0.1 ml of suspension was transferred to each well (4 × 10^4^ cells/ml) and cultured in a 37°C/5% CO_2_ incubator. Afterward, CCK-8 reagent (10 μl) was added at the 24th, 48th, 72nd, and 96th hour of incubation. Four hours later, the optical density value at 450 nm was read under a Multiskan™ FC Microplate Photometer (Thermo Scientific, U.S.A.).

### Cell invasion

Cell invasion was determined by Transwell assay. Cells transfected for 48 h were prepared into suspension (4 × 10^4^ cells/ml). The cells were transferred to apical chamber coated with Matrigel (BD, Franklin Lakes, NJ, U.S.A.). Culture medium with 10% FBS in the basolateral chamber was incubated for 24 h. The substrate and cells in apical chamber were wiped off. The membrane was washed three times using phosphate buffer saline (PBS), fixed with paraformaldehyde for 10 min, then washed three times again with double distilled water, stained with 0.5% Crystal Violet after drying. At last, invading cells were counted under a microscope in five random fields.

### Cell apoptosis

After being digested by 0.25% trypsin (Gibco) and washed twice by PBS, the transfected cells were prepared into suspension (1 × 10^6^ cells/ml) with 100 μl binding buffer. The suspension was cultured along with AnnexinV-FITC (Yisheng Biotechnology Co., Ltd., Shanghai, China) and propidium iodide (PI) for 5 min at indoor temperature in dark. Cell apoptosis was measured by an FC500MCL flow cytometer. The test was repeated for three times to take the average value.

### Western blot

The cultured cells were lysed with radio immunoprecipitation assay (RIPA) buffer (Thermo Scientific, U.S.A.) and the protein concentration was determined with bicinchoninic acid (BCA) kit (Thermo Scientific, U.S.A.). The extracted total proteins separated by 10% sodium dodecyl sulfate/polyacrylamide gel electrophoresis (SDS-PAGE) were blocked for 2 h with 5% defatted milk, then added with Sox4 protein antibody (1:1000, Abcam, U.S.A.). Glyceraldehyde-3-phosphate-dehydrogenase (GAPDH) (1:1000) was served as the internal control. Thermo Supersignal West Pico was employed to visualize the band (relative expression = protein expression/GAPDH).

### qRT-PCR

Total RNAs were isolated from the collected samples by a TRIzol kit (Invitrogen, U.S.A.), and the concentration, purity, and integrity were determined by agarose gel electrophoresis and an ultraviolet spectrophotometer. Subsequently, the RNAs were reverse-transcribed using a TaqMan™ Reverse Transcription Kit (Invitrogen, U.S.A.). The cDNAs obtained were subjected to subsequent research. PCR amplification was carried out using a PrimeScript RT Master Mix kit (Takarabo, Japan), with an amplification system containing 10 μl of SYBR qPCR Mix, 0.8 μl each of upstream and downstream primers, 2 μl of cDNAs, 0.4 μl of 50× Rox reference dye, and finally made up to 20 μl with RNase-free water. PCR conditions: pre-denaturation at 95°C for 60 s, 40 cycles of denaturation at 95°C for 30 s, annealing and extension at 60°C for 40 s. Three parallel repeating wells were designed, and all samples were repeatedly tested for three times. GADPH and U6 were served as gene and miR internal controls, respectively, and 2^−ΔΔ*c*_t_^ was used to analyze the data [17]. The 7500PCR instrument was purchased from Applied Biosystems. Primer sequences are shown in Table 1.

**Table 1:** Primer sequences

Gene	Upstream primer	Downstream primer
*LINC01694*	5′-AAACGTCGCCTTCATCCACC-3′	5′-CTGACCAACGGCTTCCTGAC-3′
*miR-340-5p*	5′-CCGTTAGTTACGATTCGAAG-3′	5′-AGGCCGCGCGTAGTGATGCAACA-3′
*Sox4*	5′-CCGAGCTGGTGCAAGACC-3′	5′-CCACACCATGAAGGCGTTC-3′
*si-LINC01694*		
*miR-340-5p-mimic*	5′-UUAUAAAGCAAUGAGACUGAUU-3′	5′-UCAGUCUCAUUGCUUUAUAAUU-3′
*miR-340-5p-inhibitor*	5′-UUCUCCGAACGUGUCACGUTT-3′	5′-ACGUGACACGUUCGUAGAATT-3′
*si-Sox4*	5′-GGACAGACGAAGAGUUUAAAG-3′	5′-UUAAACUCUUCGUCUGUCCUU-3′
*GAPDH*	5′-CCTAGGTAAACTAGACGA-3′	5′- ATTATCTGTGTCTGCATGGC-3′
*U6*	5′-AACCTTATATCGGGCGGGA-3′	5′-TTACGGCGATGCATAAT-3′

### RIP test

RIP was employed to identify the interaction or binding relationship of LINC01694 and miR-340-5p with Ago2 (Abcam, Cambridge, England), a potential binding protein in GBC-SD cells. After lysing using an EZMagna RIP kit (Millipore, Billerica, MA, U.S.A.), GBC-SD cells were incubated with protein A magnetic beads that could be conjugated to antibodies at 4°C. Six hours later, the beads were washed with washing buffer, and then cultured with 0.1% SDS/protease K (0.5 mg/ml) at 55°C for 30 min to remove proteins. Finally, the immunoprecipitated RNAs were analyzed by qRT-PCR to prove the existence of LINC01694 and miR-340-5p using specific primers.

### RNA pull-down

GBC-SD cells were transfected with biotinylated miR-340-5p-wild-type (wt), miR-340-5p-mutant (mut) and negative control (NC) (GenePharma, Shanghai, China), respectively. After 48 h, the cell lysate was incubated with M-280 Streptavidin (Invitrogen). The level of LINC01694 in the RNA complex bound to the beads was then measured by qRT-PCR.

### Dual-luciferase reporter assay

Based on the targeted binding sites predicted by the online website, the mutations of LINC01694 and Sox4 were constructed. Afterward, LINC01694 and Sox4 wild-type fragments (LINC01694-WT and Sox4-WT), LINC01694 and Sox4 mutant fragments (LINC01694-MUT and Sox4-MUT) were ligated to the psi-CHECK2 dual luciferase vector (Promega, Madison, Wisconsin, U.S.A.). Subsequently, miR-340-5p-mimics and the constructed vector were co-transfected into HEK293T cells. After 48 h of culture, luciferase activity was detected using a dual-luciferase® reporter asya system (Promega), and the results were monitored using a GloMax®20/20 Luminometer.

### Nude mouse xenograft model

GBC-SD cells were transfected with stably expressed pcDNA-3.1-LINC01694, si-LINC01694, pcDNA-3.1 vectors. Afterward, 1 × 10^6^ cells were subcutaneously injected to flank of BALB/c nude mice (4-week-old, male, five in each group), feeding for 4 weeks. The tumor volume of nude mice was measured weekly, and the calculation formula was: 0.5 × length × width^∧2^. Four weeks later, the mice were killed to collect tumor tissue and calculate the tumor weight. The present study received approval from the Animal Ethics Committee of Qilu Hospital, Shandong University and was in line with the *Guide for the Care and Use of Laboratory Animals* developed by the National Institutes of Health (NIH). Animals experiment took place in laboratory of Qilu Hospital, Shandong University. The mice were killed by carbon dioxide inhalation as follows: 100% carbon dioxide was introduced into a bedding-free cage initially containing indoor air. The lid was closed immediately to induce rapid anesthesia, and the mice died within 2.5 min.

### Statistical analysis

GraphPad 7 and SPSS20.0 were employed for building graphs and analyzing independent prognostic factors of patients, respectively. The measurement data distribution was identified by the Kolmogorov–Smirnov (K–S) test, wherein normally distributed data were expressed as mean ± standard deviation 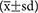, and intergroup comparison was conducted by independent samples *t* test. Counting data expressed as percentage (%) were analyzed by chi-square test (denoted by 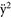). Multigroup comparison was conducted using one-way analysis of variance (ANOVA) (denoted by F). Fisher’s least significant difference-t (LSD-t) test was employed for post hoc pairwise comparison, repeated-measurement ANOVA for comparison among multiple time points (denoted by F), Bonferroni for the post hoc test. The receiver operating characteristic (ROC) curve was applied to assess the diagnostic value of LINC01694 in GBC, Pearson’s test to analyze the correlation of the genes, Kaplan–Meier (K–M) survival curve and Log-rank test to determine the total survival of patients, and multivariate Cox regression to evaluate the prognosis of patients. There was statistical difference as *P*<0.05.

## Results

**Figure 1:**
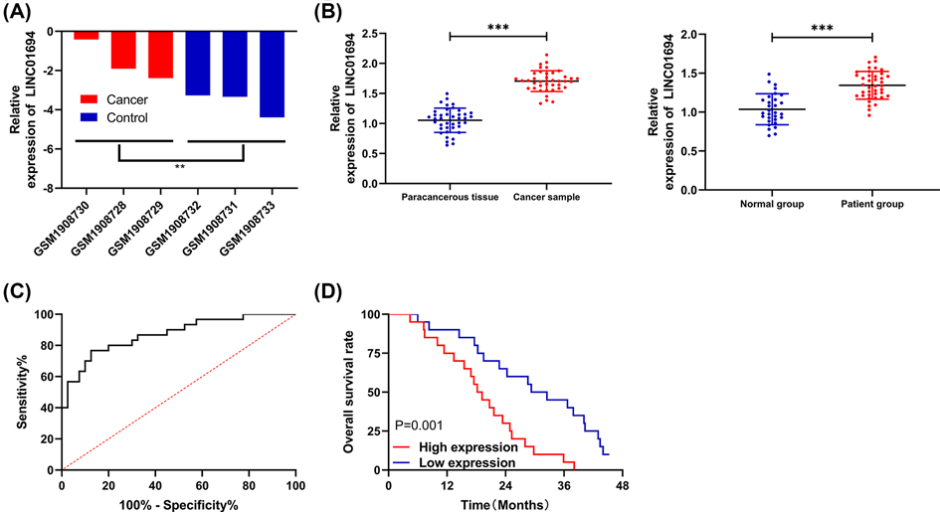
Expression of LINC01694 in patients with GBC (**A**) Relative expression of LINC01694 in GSE74048 chip. (**B**) Expression of LINC01694 in tissues and sera of patients with GBC by qRT-PCR. (**C**) AUC of LINC01694 for diagnosing GBC by ROC curve. (**D**) Survival of patients with high and low LINC01694 expression by K–M survival curve. **P<0.01, ***P<0.001. Abbreviation: AUC, area under the curve.

Mechanism diagram of the present study (Figure 1).

### Elevated expression of LINC01694 indicates poor prognosis of patients with GBC

In GSE74048 chip, the elevated expression of LINC01694 in tumor samples was revealed (Figure 1A). To confirm this, we performed qRT-PCR and found that the expression of LINC01694 was remarkably elevated in tissues and sera of patients with GBC (Figure 1B), and the area under the curve (AUC) in diagnosing GBC was 0.871 (Figure 1C). In addition, according to the median LINC01694 expression, the patients were divided into high and low expression groups, and the association between LINC01694 and pathological data was checked. Patients with high LINC01694 were more likely to develop stage III + IV and poorly differentiated GBC (Table 2). Moreover, the follow-up indicated that the patients with highly expressed LINC01694 showed a decreased total survival rate (Figure 1D). Cox regression illustrated that LINC01694 was an independent factor in the prognosis of GBC patients (Table 3).

**Table 2:** Association between LINC01694 and pathological data of patients [*n* (%)]

Factor		LINC01694		^2^	*P*
		High expression (*n*=20)	Low expression (*n*=20)		
Age				0.921	0.337
	≥60 years old [*n*=23]	13 (65.00)	10 (50.00)		
	< 60 years old [*n*=17]	7 (35.00)	10 (50.00)		
Sex				0.114	0.736
	Male [*n*=13]	6 (30.00)	7 (35.00)		
	Female [*n*=27]	14 (70.00)	13 (65.00)		
Tumor size				0.404	0.525
	≥5 cm [*n*=22]	10 (50.00)	12 (60.00)		
	<5 cm [*n*=18]	10 (50.00)	8 (40.00)		
Differentiation				8.286	0.004
	Poorly differentiated [*n*=17]	13 (65.00)	4 (20.00)		
	Moderately and highly differentiated [*n*=23]	7 (35.00)	16 (80.00)		
TNM staging				10.101	0.002
	I+II [*n*=22]	6 (30.00)	16 (80.00)		
	III+IV [*n*=18]	14 (70.00)	4 (20.00)		

**Table 3:** Cox regression

Factor	Univariate Cox regression			Multivariate Cox regression		
	*P*	HR	95% CI	*P*	HR	95% CI
Age (≥60 vs*.* <60 years)	0.325	0.715	0.367–1.394			
Sex (Male vs*.* Female)	0.787	0.909	0.457–1.81			
Tumor size (≥ 5 vs. < 5 cm)	0.097	1.732	0.906–3.311			
Differentiation (Lowly differentiated vs. Moderately+Highly differentiated)	0.004	0.354	0.175–0.717	0.138	0.539	0.238–1.221
TNM staging (I+II vs. III+IV)	0.006	2.556	1.304–5.012	0.013	0.402	0.195–0.827
LINC01694 (Yes vs. No)	0.002	0.318	0.153–0.661	0.022	2.242	1.126–4.465

Abbreviation: CI, confidence interval; HR, hazard ratio.

### Knockdown of LINC01694 inhibits growth of GBC cells

**Figure 2:**
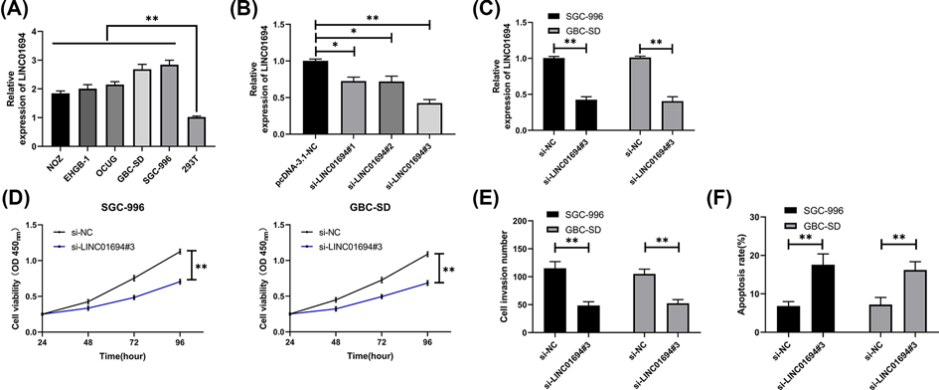
Knockdown of LINC01694 inhibits the growth of GBC cells (**A**) Expression of LINC01694 in GBC cells by qRT-PCR. (**B**) Relative expression of LINC01694 in vectors after transfection by qRT-PCR. (**C**) Expression in GBC cells transfected with si-LINC01694#3 by qRT-PCR. (**D**) Growth of GBC cells after transfection of si-LINC01694#3 by CCK-8. (**E**) Invasion of GBC cells after transfection of si-LINC01694#3 by Transwell. (**F**) Apoptotic rate of GBC cells after transfection of si-LINC01694#3 by flow cytometry. *P<0.05, **P<0.01.

The expression of LINC01694 increased significantly in GBC cell lines (Figure 2A). To analyze the effects of LINC01694 on the growth of GBC cells, we established three LINC01694 inhibitors (si-LINC01694 # 1, 2, 3), and si-LINC01694#3 was found to have the most obvious inhibitory effect (Figure 2B), so it was transfected into SGC-996 and GBC-SD cell lines (Figure 2C). According to CCK-8 and Transwell, knockdown of LINC01694 remarkably weakened cell proliferation (Figure 2D) and invasion (Figure 2E) compared with pcDNA-3.1-NC. However, apoptosis test showed that the apoptotic rate in LINC01694 knockdown cells was elevated (Figure 2F).

### LINC01694 acts as a sponge to regulate miR-340-5p

**Figure 3:**
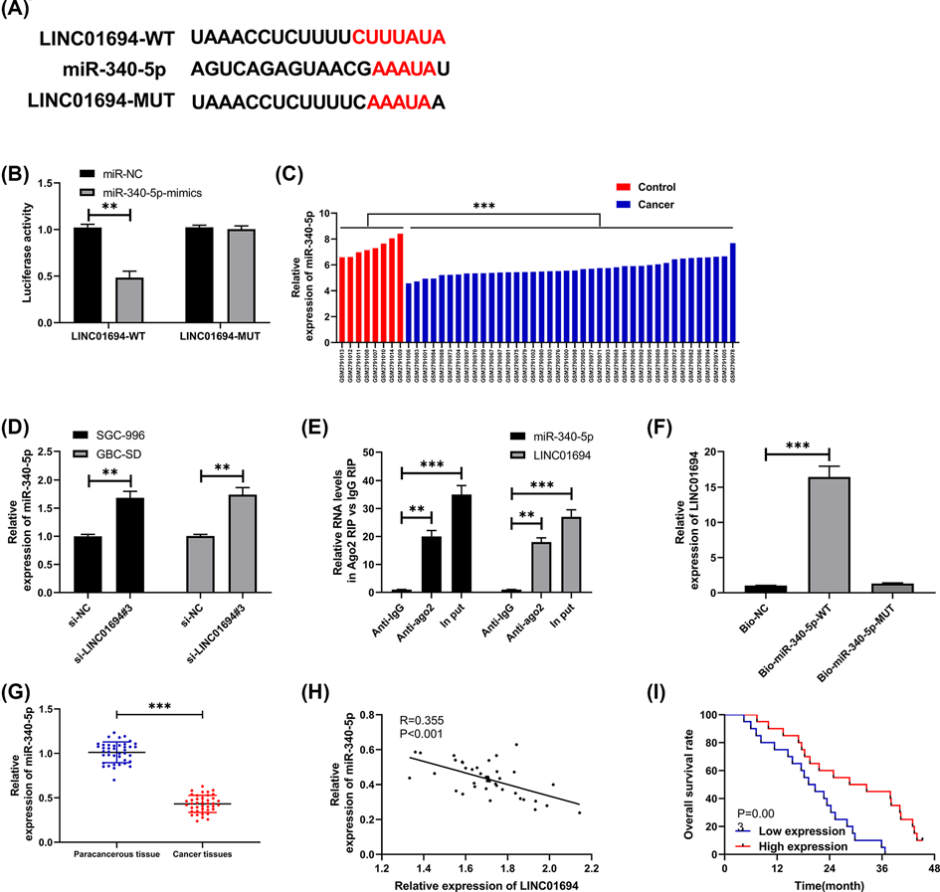
LINC01694 regulates miR-340-5p (**A**) Bioinformatics analysis reveals that LINC01694 shares targeted binding loci with miR-340-5p. (**B**) DLR assay confirms the targeting relation between LINC01694 and miR-340-5p. (**C**) MiR-340-5p expression in GBC cells through GSE104165 chip. (**D**) Expression of miR-340-5p after knockdown of LINC01694 by qRT-PCR. (**E**) RIP test confirms that LINC01694 is bound to miR-340-5p and Ago2. (**F**) RNA pull-down confirms that LINC01694 is pulled down by miR-340-5p-wt. (**G**) MiR-340-5p is lowly expressed in GBC tissues by qRT-PCR. (**H**) Correlation between miR-340-5p and LINC01694 in GBC tissues by Pearson’s test. (**I**) Total survival rate in patients with high and low expression of miR-340-5p by K–M survival curve. **P<0.01, ***P<0.001.

We predicted the targeting relation between LINC01694 and miRs to display the relevant mechanism of LINC01694, and the results demonstrated that LINC01694 shared targeted binding loci with miR-340-5p (Figure 3A), then we found miR-340-5p was lowly expressed in GBC through GSE104165 (Figure 3C). Moreover, dual-luciferase reporter (DLR) assay confirmed that miR-340-5p-mimics inhibited the fluorescence activity of LINC01694-wt (Figure 3B) and down-regulating LINC01694 elevated miR-340-5p in cells (Figure 3D). In addition, RIP test and RNA pull-down verified that LINC01694 was bound to miR-340-5p. RIP test exhibited that the levels of LINC01694 and miR-340-5p precipitated by Ago2 antibody were significantly higher than those precipitated by IgG antibody (Figure 3E). While RNA pull-down found that LINC01694 was pulled down by biotin-labeled miR-340-5p-wt, but no such effect was induced by miR-340-5p-mut (Figure 3F). Furthermore, miR-340-5p was lowly expressed in GBC tissues (Figure 3G). According to correlation analysis, LINC01694 was negatively correlated with the relative expression of miR-340-5p (Figure 3H), and the total survival rate of patients with low miR-340-5p was remarkably reduced (Figure 3I).

### MiR-340-5p regulates Sox4 targetedly

**Figure 4:**
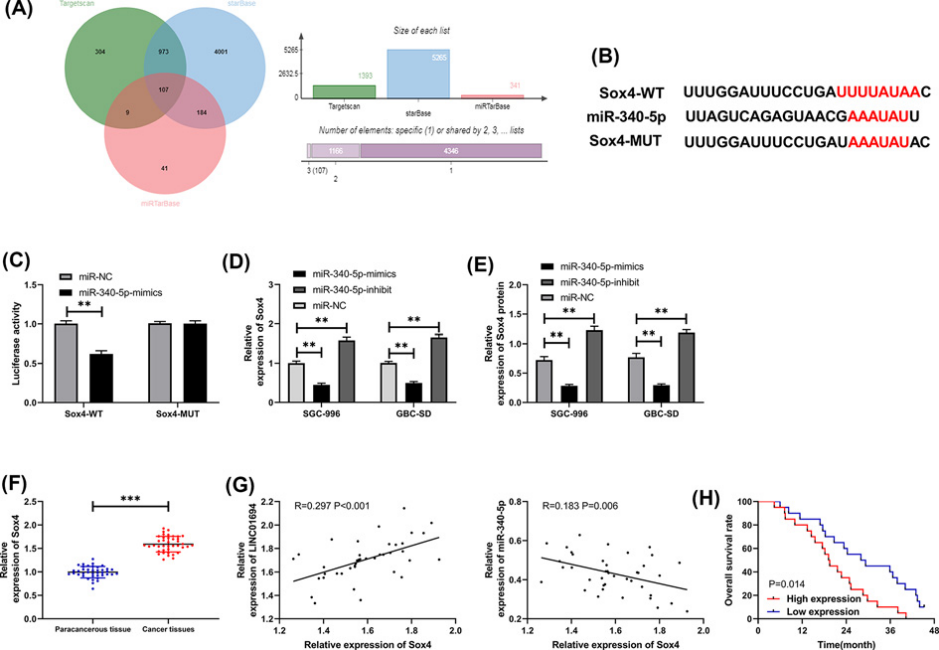
Association between miR-340-5p and Sox4 (**A**) Prediction of downstream target genes for miR-340-5p by TargetScan, starBase and miRTarBase. (**B**) Targeted binding loci between miR-340-5p and Sox4. (**C**) DLR assay on miR-340-5p and Sox4. (**D,E**) Levels of Sox4 mRNA and protein in cells transfected with miR-340-5p-mimics/inhibit by qRT-PCR and WB. (**F**) Sox4 expression in GBC tissues by qRT-PCR. (**G**) Correlation between Sox4 and miR-340-5p and LINC01694 by Pearson’s test. (**H**) Total survival rate in patients with high and low expression of Sox4 by K–M survival curve. **P<0.01, ***P<0.001. Abbreviations: WB, Western blot.

Prediction for the target genes uncovered the role of Sox4 as a potential target gene of miR-340-5p (Figure 4A,B). We transfected vectors with different expression of miR-340-5p into GBC cells and measured the expression of Sox4. The relative expression of Sox4 in cells transfected with miR-340-5p-mimics was decreased, while that in cells transfected with miR-340-5p-inhibit was increased (Figure 4D,E). As shown by DLR assay, miR-340-5p-mimcs suppressed the luciferase activity of Sox4-wt (Figure 4C). In addition, the expression of Sox4 was elevated in GBC tissues (Figure 4F). And Sox4 was positively correlated with LINC01694 and negatively correlated with miR-340-5p (Figure 4G). The total survival rate was remarkably reduced in patients with high Sox4 expression (Figure 4H).

### LINC01694 acts as a sponge to regulate miR-340-5p/Sox4 axis and affect growth of GBC cells

**Figure 5:**
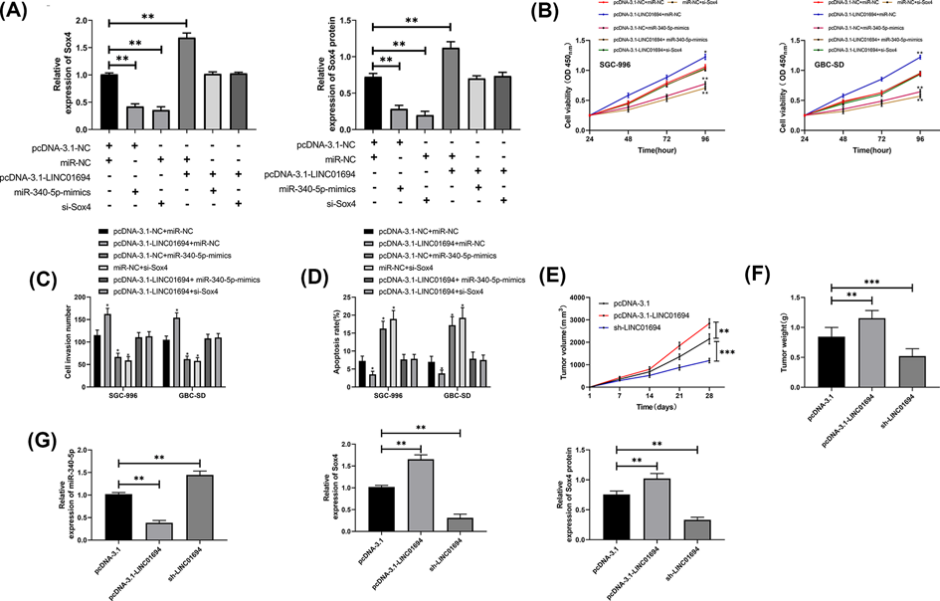
LINC01694 affects growth of GBC cells by regulating miR-340-5p/Sox4 axis (**A**) Relative expression of Sox4 mRNA and protein in co-transfected cells by qRT-PCR and WB. (**B**) Cell proliferation after transfection by CCK-8. (**C**) Number of invading cells after transfection by Transwell. (**D**) Changes of apoptotic rate after transfection by flow cytometry. (**E**) Tumor volume changes in nude mice after transfection of overexpression and knockdown of LINC01694 vectors. (**F**) Tumor weight of nude mice after 28 days. (**G**) Relative expression of miR-340-5p and Sox4 protein/mRNA in tumor tissues of nude mice by qRT-PCR and WB. *P<0.05, **P<0.01, ***P<0.001.

Whether LINC01694 affected the growth of GBC cells via miR-340-5p/Sox4 axis was explored in our study. Cells were transfected with pcDNA-3.1-NC + miR-NC, pcDNA-3.1-LINC01694 + miR-NC, pcDNA-3.1-NC + miR-340-5p-mimics, miR-NC + si-Sox4, pcDNA-3.1-LINC01694 + miR-340-5p-mimics, pcDNA-3.1-LINC01694 + si-Sox4, respectively. It turned out that Sox4 was elevated in cells transfected with pcDNA-3.1-LINC01694, and was suppressed after transfection of pcDNA-3.1-LINC01694 + si-Sox4 or pcDNA-3.1-LINC01694 + miR-340-5p-mimics (Figure 5A). Moreover, cell proliferation (Figure 5B) and invasion (Figure 5C) were significantly enhanced and the apoptosis (Figure 5D) was decreased after transfection of pcDNA-3.1-LINC01694 alone. However, the outcome was reversed after its co-transfection with either miR-340-5p-mimics or si-Sox4. Nude mouse xenograft models revealed that in nude mice injected with cells transfected with HBLV-h-LINC01694, the tumor volume and mass increased significantly (Figure 5E,F), miR-340-5p decreased, and the relative expression of Sox4 mRNA and protein increased (Figure 5G). However, the above outcomes were reversed in mice injected with sh-LINC01694 transfected cells.

## Discussion

**Figure 6:**
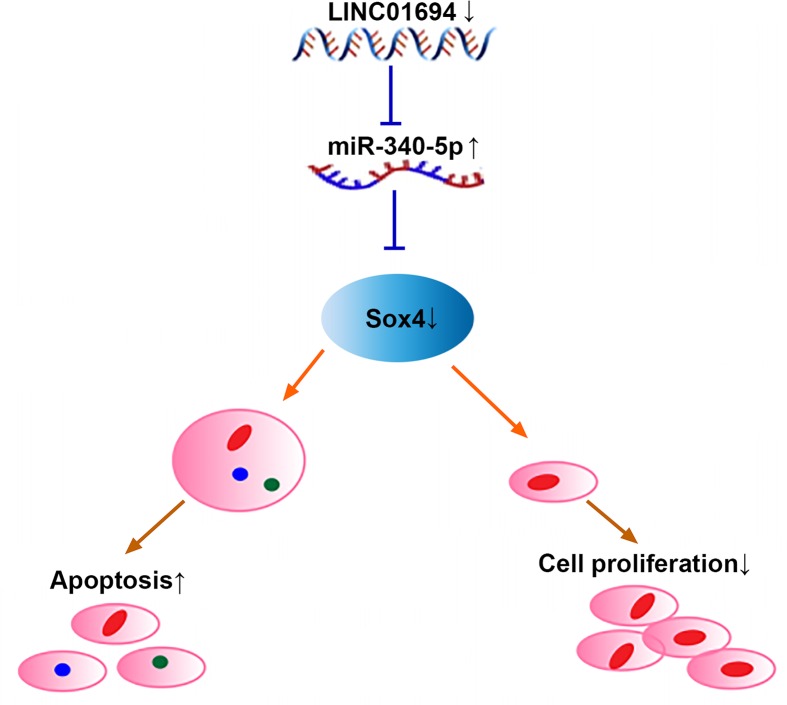
Mechanism diagram of the present study

GBC is a digestive tract tumor with high malignancy and unsatisfactory survival and prognosis [18]. In our study, elevated expression of LINC01694 was revealed in GBC, which indicated the poor prognosis of patients. In addition, patients with high expression are more likely to develop stage III–IV and poorly differentiated GBC. We also found that down-regulation of LINC01694 inhibited growth and metastasis of GBC cells by mediating miR-340-5p/Sox4 axis (Figure 6), suggesting that LINC01694 is expected to be a potential target for the treatment of GBC.

LINC01694 is a newly discovered lncRNA (>200 nt [19]), which has never been studied previously. In the present study, we revealed the elevated expression of LINC01694 in GBC through chip screening, suggesting that LINC01694 was expected to be a prognostic/diagnostic marker for GBC. We first observed the expression of LINC01694 in patients. Due to the hidden location of the gallbladder, biopsy requires the assistance of imaging technologies, while serum sample detection has the characteristics of reducing invasive injury and facilitating collection [20]. Our findings indicated that the LINC01694 expression was up-regulated in both tissues and sera of patients with GBC, they were also positively correlated. In addition, ROC curve revealed the high value of expression of serum LINC01694 in diagnosis of GBC, suggesting that serum lncRNA might be a potential marker for GBC. The low survival rate and absence of prognostic markers are knotty problems in GBC treatment. The patients were allocated into groups to explore the correlation between LINC01694 and survival and prognosis. The survival rate of patients with high LINC01694 was significantly reduced. And Cox regression found that the elevated expression of LINC01694 indicated poor prognosis of the patients. Therefore, we preliminarily determined the clinical value of LINC01694 in GBC, but the relevant mechanisms were not clear.

In recent years, competitive endogenous RNA (ceRNA) has been discussed in various fields [21,22]. It is proposed mainly to describe the interaction between lncRNA and its coding genes [23]. LncRNA acts as a natural miR sponge to inhibit its target genes through competitive binding to miRs. For example, Mou et al. [24] pointed out that lncRNA-activated by transforming growth factor β (lncRNA-ATB) acted as a ceRNA to promote Yes-Associated Protein 1 (YAP1) through sponging miR-590-5p in malignant melanoma. Through online software prediction, we found that there was a potential targeted binding possibility between LINC01694 and miR-340-5p. Besides, DLR assay, RIP test, and RNA pull-down proved that LINC01694 could be served as a sponge to regulate miR-340-5p.MiR-340-5p is a tumor suppressor lowly expressed in breast cancer, thyroid cancer, and osteosarcoma. However, its expression in GBC has not been determined yet. Therefore, GBC-related miR chips were screened out through GEO database, and the low expression of miR-340-5p in GBC was revealed. We also discovered the suppressed expression of miR-340-5p in GBC tissues and the survival rate of patients was reduced. In addition, there was a negative correlation between miR-340-5p and LINC01694, suggesting the targeting relation between them.

Regulating downstream target genes to participate in biological processes is one of the mechanisms of miRs [25]. We identified that Sox4 was a potential target gene of miR-340-5p using an online prediction software. Sox4 is a coding protein regulating embryonic development and cell apoptosis [26]. And in Wang et al.’s [27] study, it was found that Sox4 was elevated in patients with GBC and was an independent factor affecting the prognosis. In the present study, up-regulated Sox4 in cancerous tissues led to the low survival rate of patients. In addition, correlation analysis uncovered the positive correlation between Sox4 and LINC01694 and the negative correlation between Sox4 and miR-340-5p. The above studies suggested the regulatory mechanism between LINC01694 and miR-340-5p and Sox4. Subsequently, the growth of co-transfected cells and Xenograft model in nude mice illustrated that LINC01694 participated in the growth of GBC cells via regulating miR-340-5p/Sox4 axis.

In the present study, we confirmed the value and related mechanism of LINC01694 in GBC. However, there are still several limitations. First, the small size of samples in our study may lead to bias in the conclusion. Second, although we have confirmed that LINC01694 may be a potential target for GBC, the specific dosage regimen or treatment scheme have not been introduced. Finally, it is not clear whether LINC01694 participates in the development of GBC in other ways. Therefore, we will enlarge our sample size, carry out follow-ups and bioinformatics prediction to address our research deficiencies.

## Conclusion

In conclusion, LINC01694 expression is elevated in GBC by regulating miR-340-5p/Sox4 axis, which indicates the poor prognosis of the patients.

## Abbreviations

Ago2: argonaute RISC catalytic component 2
ANOVA: analysis of variance
CCK-8: cell counting kit-8
ceRNA: competitive endogenous RNA
DLR: dual-luciferase reporter
DMEM: Dulbecco’s modified Eagle’s medium
FBS: fetal bovine serum
GAPDH: glyceraldehyde-3-phosphate-dehydrogenase
GBC: gallbladder cancer
GEO: Gene Expression Omnibus
LINC01694: long intergenic non-protein coding RNA 1694
lncRNA: long non-coding RNA
NC: negative control
ncRNA: non-coding RNA
PBS: phosphate buffer saline
qRT-PCR: quantitative real-time polymerase chain reaction
RIP: RNA immunoprecipitation
ROC: receiver operating characteristic
SDS: sodium dodecyl sulfate
